# Filled Pauses Produced by Autistic Adults Differ in Prosodic Realisation, but not Rate or Lexical Type

**DOI:** 10.1007/s10803-023-06000-y

**Published:** 2023-05-03

**Authors:** Simon Wehrle, Martine Grice, Kai Vogeley

**Affiliations:** 1 Institut für Linguistik-Phonetik, University of Cologne, Cologne, Germany; 2 Department of Psychiatry, University Hospital Cologne, Medical Faculty, University of Cologne, Cologne, Germany; 3 Cognitive Neuroscience, Institute of Neuroscience and Medicine (INM-3), Research Center Jülich, Jülich, Germany

**Keywords:** Autism spectrum disorder, Filled pause, Hesitation, Prosody, Conversation

## Abstract

We examined the use of filled pauses in conversations between homogeneous pairs of autistic and non-autistic adults. A corpus of semi-spontaneous speech was used to analyse the rate, lexical type (nasal “uhm” or non-nasal “uh”), and prosodic realisation (rising, level or falling) of filled pauses. We used Bayesian modelling for statistical analysis. We found an identical rate of filled pauses and an equivalent preference of “uhm” over “uh” across groups, but also a robust group-level difference regarding the intonational realisation of filled pauses: non-autistic controls produced a considerably higher proportion of filled pause tokens realised with the canonical level pitch contour than autistic speakers. Despite the fact that filled pauses are a frequent and impactful part of speech, previous work on their conversational use in autism spectrum disorder (ASD) is limited. Our account is the first to analyse the intonational realisation of filled pauses in ASD and the first to investigate conversations between autistic adults in this context. Our results on rate and lexical type can help to contextualise previous research, while the novel findings on intonational realisation set the stage for future investigations.

**Supplementary Information:**

The online version contains supplementary material available at 10.1007/s10803-023-06000-y.

## Introduction

Filled pauses such as “uh” or “uhm” are a ubiquitous feature of spoken interaction. They are typically used to signal hesitation or uncertainty, and are intended to help the current speaker hold the floor or, sometimes, to take over the floor from the interlocutor (Belz, 2021; Beňuš, 2009; Fischer, 2000; Schettino, 2019; Shriberg, 2001; Ward, 2006). Filled pauses are rarely produced consciously and deliberately.

Producing filled pauses has often been judged and perceived to be undesirable, with certain educational and training settings actually aiming to eradicate their use, at least in formal, monologic speech (Erard, 2008; Fischer, 2013; Fox Tree, 2002; Niebuhr & Fischer, 2019; O’Connell & Kowal, 2004; Smith & Clark, 1993; Ward, 2019). Although a higher rate of filled pauses can lead to more negative judgements in the specific case of public speaking (Niebuhr & Fischer, 2019), filled pauses in dialogue actually facilitate understanding and the flow of conversation. They serve a range of crucial communicative functions, e.g. signalling politeness and attention or foreshadowing the duration and informativeness of upcoming utterances, which aids in the planning of complex utterances (Corley & Hartsuiker, 2003; Fox Tree, 2001; Fruehwald, 2016; Levinson, 1983; Niebuhr & Fischer, 2019; Rose, 2017; Schegloff, 2010; Strangert, 1991; Watanabe et al., 2008).

We will focus on the two most common types of filled pauses (by far), those realised either with only a central vowel (*uh*) or a central vowel followed by a nasal (*uhm*). Although similar in segmental form, a number of studies have found important differences between *uh* and *uhm*, suggesting e.g. that *uh* is perceived more negatively than *uhm* (Niebuhr & Fischer, 2019) and that *uhm* is not only more frequent than *uh*, but is continuing to gain ground in an ongoing process of linguistic change over time (Fruehwald, 2016; Wieling et al., 2016). Some authors have further proposed that *uhm* might be functionally different from *uh*. *Uhm* not only seems to reliably cue longer silent pauses than *uh* (Clark & Fox Tree, 2002; Fox Tree, 2001)—a finding which we recently extended and qualified in the context of ASD (Wehrle et al., 2023a)—but it has also been suggested that *uhm* might be a more specifically listener-oriented conversational signal than *uh* (Gorman et al., 2016; Irvine et al., 2016; McGregor & Hadden, 2020).

It is important to note at this point that all of these specific aspects of filled pause production can only safely be assumed to apply to West Germanic languages, as most studies used data from German, English or Dutch (for which results are very similar). A number of studies from other language families show that, while a distinction between two filled pause types—one consisting of only a vowel and the other with the addition of a final nasal—is very common, there are differences in their exact phonetic realisation, especially in terms of vowel quality (Anansiripinyo & Onsuwan, 2019; Di Napoli, 2020; Kosmala & Crible, 2022; Nguyễn, 2015; Schettino, 2019; Yuan et al., 2016). There also seems to be a differential (possibly increased) use of other forms of hesitation such as repetition and prolongation in other languages, and particularly in tone languages (Betz et al., 2017; Lee et al., 2004; Tseng, 2003).

Regarding prosodic realisation, there is abundant, cross-linguistic evidence that filled pauses are typically produced with flat or level intonation contours, and that they tend to be relatively low in pitch (Adell et al., 2010; Belz & Reichel, 2015; O’Shaughnessy, 1992; Shriberg & Lickley, 1993). Our study is the first to consider prosodic aspects of filled pauses in the context of autism spectrum disorder (ASD).

### Previous Work on Filled Pauses in Autism Spectrum Disorder

Research on the use of filled pauses by speakers on the autism spectrum is limited, but growing. We are aware of eight previous studies focussing on filled pauses in ASD, none of which analysed conversations between autistic adults (as we do here). Seven out of these eight studies analysed the speech of children or adolescents (Gorman et al., 2016; Irvine et al., 2016; Jones et al., 2022; Lunsford et al., 2010; McGregor & Hadden, 2020; Parish-Morris et al., 2017; Suh et al., 2014), while one analysed the speech of autistic adults interacting with a—presumably non-autistic—experimenter (Lake et al., 2011). Most studies analysed speech that was either monologic or produced in the context of structured interviews with a trained professional (with the exception of Jones et al., 2022, who used semi-structured double interviews), in many cases through use of the autism diagnostic observation schedule (ADOS) (Lord et al., 2000).

All studies but one (Suh et al., 2014) found differences between the filled pause productions of autistic and non-autistic participants. Of these, the only previous study on filled pauses in the speech of adults on the autism spectrum found a lower rate of filled pauses across lexical types (*uh* and *uhm*), while the remaining six studies considering children all report a lower proportion (or rate) of only *uhm*, but not *uh*, in the speech of autistic as compared to non-autistic subjects.

These findings have led to a suggestion in some of the works cited above that the nasal filled pause type *uhm* might have a distinctly listener-oriented function, and that the pattern of a reduced production of *uhm*, specifically, might help to distinguish ASD from related diagnoses (Gorman et al., 2016) and serve as a pragmatic (Irvine et al., 2016) or even clinical marker (McGregor & Hadden, 2020). Gorman et al. (2016) further suggest that “fillers (…) may be a useful target for intervention” (p. 862). Such speculations have to be treated with caution, however. Not only is the amount of evidence rather limited to date, especially when taking into account the serious and pertinent issue of publication bias (whereby studies that find a “significant” effect are vastly more likely to be published than those that do not; DeVito & Goldacre, 2019; Easterbrook et al., 1991; John et al., 2012; Sterling, 1959). More specifically, the relevant pattern of a reduced use of *uhm* (specifically and exclusively) does not seem to hold true for autistic adults, as suggested by the only relevant previous study (Lake et al., 2011) as well as the findings presented in this paper.

### Current Study

With the current study, we aim to make a novel contribution to the literature on filled pause production in ASD by (1) analysing conversations between autistic adults and (2) considering the prosodic realisation of filled pauses in the context of ASD.

We recorded pairs of autistic speakers (ASD–ASD), or disposition-matched dyads, rather than mixed dyads (ASD–CTR) for two main reasons. First, there is a drastic lack of research on communication in ASD based on data from matched rather than mixed dyads, both in general and for the specific area of filled pauses in ASD (all previous studies having investigated mixed dyads). Second, investigating the behaviour of disposition-matched dyads seems to us the most promising way to gain insights into what we might justifiably call an “autistic conversation style” (Bolis et al., 2017; Davis & Crompton, 2021; Milton, 2012; Mitchell et al., 2021; Rifai et al., 2022; Sheppard et al., 2016).

Prosody is a particularly important and potentially distinctive aspect of communication in ASD, as pointed out in a wealth of previous research (e.g. Krüger, 2018; McCann & Peppé, 2003; Paul et al., 2005; Wehrle et al., 2020, 2022). Although there is a substantial amount of previous research on the prosodic realisation of filled pauses in the general population, this aspect has not been considered in work on ASD to date. Given that previous findings point to a very clear cross-linguistic tendency for filled pauses being produced with flat or level intonation, we focus here mainly on investigating whether there is any deviation from this convention in our data set. Our own previous work on backchannels—listener signals such as “mmhm” or “okay” which are closely related to filled pauses in form and function—in the same corpus of speech as investigated here has revealed intriguing differences between autistic and non-autistic speakers in terms of prosodic realisation (Wehrle, 2021). In combination with the fact that related work has shown the importance of the precise prosodic realisation of backchannels for listener judgements and mutual understanding (Cutrone, 2014; Ha et al., 2016; Li, 2006; Wehrle & Grice, 2019; Wehrle et al., 2018), we can speculate that the exact prosodic realisation of filled pauses may be similarly impactful.

Although differences in terms of speakers, conversational context and language prevent us from directly extrapolating from previous findings to our own analyses, we might reasonably predict (1) that fewer filled pauses (especially of the *uhm* type) will be produced in conversations between autistic adults and (2) that the prosodic realisation of filled pauses will differ between the ASD and the CTR group.

## Method

### Participants

We recorded 28 monolingual native speakers of German engaged in semi-structured conversation, of which 14 had been diagnosed with Asperger syndrome (corresponding to ICD-10: F84.5; see World Health Organization, 1992) and were recruited in the Autism Outpatient Clinic at the Department of Psychiatry of the University of Cologne (Germany). As part of a systematic assessment implemented in the clinic, diagnoses were made independently by two different specialized clinicians corresponding to ICD-10 criteria, and supplemented by an extensive neuropsychological assessment. Participants from the control group (CTR) were recruited from the general population specifically for this study. All subjects were paid 10 EUR for participation. We ascertained that participants had not been acquainted with each other before the start of the experiment (although some participants in the ASD group may have crossed paths in the context of the autism outpatient clinic). Participants were grouped into homogeneous, disposition-matched dyads (7 ASD–ASD, 7 CTR–CTR).

All participants completed the German version of the autism-spectrum quotient (AQ) questionnaire (Baron-Cohen et al., 2001). All participants also completed the *Wortschatztest WST* (Schmidt & Metzler, 1992), a standardised, receptive German vocabulary test that exhibits a high correlation with verbal as well as general intelligence (Satzger et al., 2002).

Participants from the CTR group were matched as closely as possible to the ASD group for age, verbal IQ and gender, but some minor differences remained. Participants from the ASD group were on average slightly older (mean = 44; range: 31–55) than participants from the CTR group (mean = 37; range: 29–54). However, there was extensive overlap between groups and there is no reason to assume that such a relatively small age difference would have any pertinent effects on filled pause production. The ASD group also had a slightly higher average verbal IQ score (mean = 118; range: 101–143) than the CTR group (mean = 106; range: 99–118). Here, too, there was considerable overlap between groups, and there is no reason to assume that this difference should have a meaningful impact on results. The gender ratio was similar, but not identical across groups. The ASD group contained 4 females and 10 males, whereas the CTR group contained 3 females and 11 males. This entails that dialogues took place in the ASD group between 1 all-female dyad, 2 mixed dyads and 4 all-male dyads, and in the CTR group between 3 mixed dyads and 4 all-male dyads.

As expected, there was a clear difference in AQ scores between groups, with a far higher average score in the ASD group (mean = 41.9; range = 35–46) than in the CTR group (mean = 16.1; range: 11–26), and no overlap at all between subjects from both groups. All autistic participants scored above and all non-autistic participants scored below the commonly suggested clinical threshold of 32 (Ashwood et al., 2016; Baron-Cohen et al., 2001). Bayesian modelling provides unambiguous evidence for the group difference in AQ scores, and also confirms that the differences in age and verbal IQ are small but robust (for details see section Statistical Analysis and the *OSF* repository at https://osf.io/6zu4g/). Table 1 shows summary statistics for gender, age, verbal IQ and AQ.

**Table 1:** Subject information by group (SD = standard deviation)

Gender (n)		Age		Verbal IQ		AQ	
Female	Male	Mean	SD	Mean	SD	Mean	SD
ASD 4	10	43.6	6.7	118.1	12.0	41.9	3.1
CTR 3	11	36.5	7.6	105.8	5.8	16.1	4.5

All aspects of the study have been approved by the local ethics committee of the Medical Faculty at the University of Cologne and were performed in accordance with the ethical standards laid down in the 1964 Declaration of Helsinki and its later amendments. All participants gave their written informed consent prior to participating in the experiment.

### Material

We used Map Tasks to elicit semi-structured conversations (Anderson et al., 1984, 1991). Participants were recorded in pairs (dyads). After filling in a number of forms and the questionnaires listed above, participants received written instructions for the task and entered a recording booth. Each participant was presented with a simple map containing nine landmark items in the form of small pictures (materials adapted from Grice & Savino, 2003, and optimised for prosodic analysis). Only one of the two participants (the instruction giver) had a route printed on their map. The experimental task was for the instruction follower to transfer this route to their own map by exchanging information with the instruction giver. (We will report on differences between instruction givers and followers only where they are informative beyond the general analysis, i.e. only for the rate of filled pauses.) During this entire process, an opaque screen was placed between participants, meaning they could not establish visual contact and had to solve the task by means of oral communication alone. The roles of instruction giver and instruction follower were assigned randomly. Upon completion of the first task, subjects received a new set of maps and their roles were switched. The session ended once the second Map Task was completed.

Conversations were recorded in a sound-proof booth at the Department of Phonetics, University of Cologne. We used two head-mounted microphones (AKG C420L) connected through an audio-interface (PreSonus AudioBox 22VSL) to a PC running Adobe Audition. The sample rate was 44,100 Hz (16 bit). We only included recorded dialogue from the start to the end of each task in all analyses, in order to achieve a greater degree of comparability regarding conversational context. The total duration of analysed dialogue is 4 h and 44 min. The mean dialogue duration is 20 min and 19 s (SD = 12′32′′; see S1 Table in the Supplementary Information for more detail).

### Data and Processing

The corpus under investigation contains 1027 filled pause tokens in total. Filled pauses were defined as all hesitations roughly of the form “äh” or “ähm” in German. All tokens including a final nasal were included in the *uhm* category and all tokens without a nasal were included in the *uh* category (we use the written form < uh(m) > rather than < äh(m) > to remain consistent with the terminology used in most previous research). We included tokens with slightly different vowel qualities that were clearly identical in function and comparable in form. We also included a very small number of tokens that were realised with only a nasal (/m/) in the *uhm* category, since in practice it was very difficult to determine a threshold for distinguishing realisations with short, reduced vowels (which can also be nasalised) followed by a nasal from those consisting of nothing but a nasal.

For prosodic analysis, we firstly hand-corrected and smoothed all tokens using *Praat* (version 6.1.09) (Boersma & Weenink, 2020) and *mausmooth* (Cangemi, 2015). We then used a custom *Praat* script to extract pitch values at 10% and 90% of token duration and calculated the difference between those values in semitones (ST; with a reference value of 1 Hz), with positive values indicating pitch rises and negative values indicating falls (cf. Ha et al., 2016; Wehrle, 2021). We used values at 10% and 90% of token duration (rather than the very first and last values) in order to minimise possible effects of microprosody and glottalization that are known to occur at the extreme edges of syllables. If there was no pitch information available at either 10% or 90% (usually because non-modal voice quality was used), the point of extraction was moved by 10%, yielding e.g. 20–90% or 10–80% windows. This procedure was repeated up to a maximum of 40% at the beginning and 70% at the end. The majority of pitch values (65%) was extracted within 20% of start duration and 80% of end duration. We finally verified all extracted values through comparison with the original extracted token and the smoothed pitch contour, and excluded any tokens that were unsuitable for intonational analysis (due to spurious or missing data). Note that there were no major inflection points between the beginning and end of the pitch contour in all tokens. In other words, all filled pauses were either produced with level pitch or consisted of simple (essentially monotonic) rises or falls. Additionally, we used Shannon entropy to quantify the diversity of pitch contours used in the production of filled pauses (cf. Wehrle, 2021).

### Statistical Analysis

For statistical analysis, we used Bayesian multilevel linear models implemented in the modelling language *Stan* (version 2.29) (Stan Development Team, 2022) via the package *brms* (version 2.16.3) (Bürkner, 2017) for the statistical computing language *R* (version 4.1.2) (R Core Team, 2022), which we used in the software *RStudio* (version 2021.09.1) (RStudio Team, 2021).

Analysis and presentation of Bayesian modelling broadly follows the example of Franke and Roettger (2019), but is also informed by a number of other tutorials (McElreath, 2020; Vasishth et al., 2018; Winter & Bürkner, 2021). We report expected values (β) under the posterior distribution and their 95% credible intervals (CIs). We also report the posterior probability that a difference δ is greater than zero. In essence, a 95% CI represents the range within which we expect an effect to fall with a probability of 95%. We consider these credible intervals in and of themselves as the most relevant outcome of Bayesian modelling. For comparability with conventional null-hypothesis significance testing and reporting practices, it may be helpful for readers to assume that if (1) zero is (by a reasonably clear margin) not included in the 95% CI of δ and (2) the posterior *P* (δ > 0) is close to one, the model provides (strong) support for a given hypothesis. Please note, however, that a dichotomous distinction between significant and non-significant effects is explicitly not required (or, to our minds, desirable) in the framework of Bayesian inferential statistics.

We used regularising weakly informative priors for all models (Lemoine, 2019) and performed posterior predictive checks with the packages *brms* (version 2.16.3) (Bürkner, 2017) and *bayesplot* (version 1.8.1) (Gabry & Mahr, 2021) in order to verify that the priors were suited to the data set. Unless otherwise specified, four sampling chains ran for 4000 iterations with a warm-up period of 2000 iterations for each model. Besides the packages for Bayesian modelling, we made extensive use of the packages included in the *tidyverse* collection for performing data import, tidying, manipulation, visualisation, and programming (Wickham et al., 2019).

In reporting experimental results in this article, Bayesian inference is used in the spirit of complementing, not superseding the descriptive, exploratory analysis that we consider to be at the heart of this work. We emphasise a fully transparent analysis aiming to provide a comprehensive understanding of experimental results first and foremost through detailed description and the extensive use of data visualisation (Anscombe, 1973; Matejka & Fitzmaurice, 2017). Therefore, not all details of Bayesian modelling are reported for all analyses here, but all information can be found in the accompanying *OSF* repository at https://osf.io/6zu4g/.

We are also committed to an in-depth analysis appropriately accounting for individual- and dyad-specific behaviour (cf. Bruggeman et al., 2017; Cangemi et al., 2015, 2016; Wehrle et al., 2023b). The importance of considering scientific data at the level of the individual and/or dyad is of particular relevance in the context of studies on autism spectrum disorder, given the characteristically high degree of variability in groups of autistic individuals. In the following, we report results by speaker for all analyses and only additionally discuss results at the dyad level where they were found to be informative beyond the individual-specific analysis (i.e. only for rate of filled pauses; all additional information can be found in the accompanying repository).

## Results

We will first present results on the rate and type of filled pauses, and then discuss prosodic aspects. The average duration of filled pauses was very similar across groups (ASD: 423 ms; CTR: 456 ms), with a grand mean of 444 ms (SD = 247), and will therefore not be considered in any more detail in the following.

### Rate of Filled Pauses

**Fig. 1:**
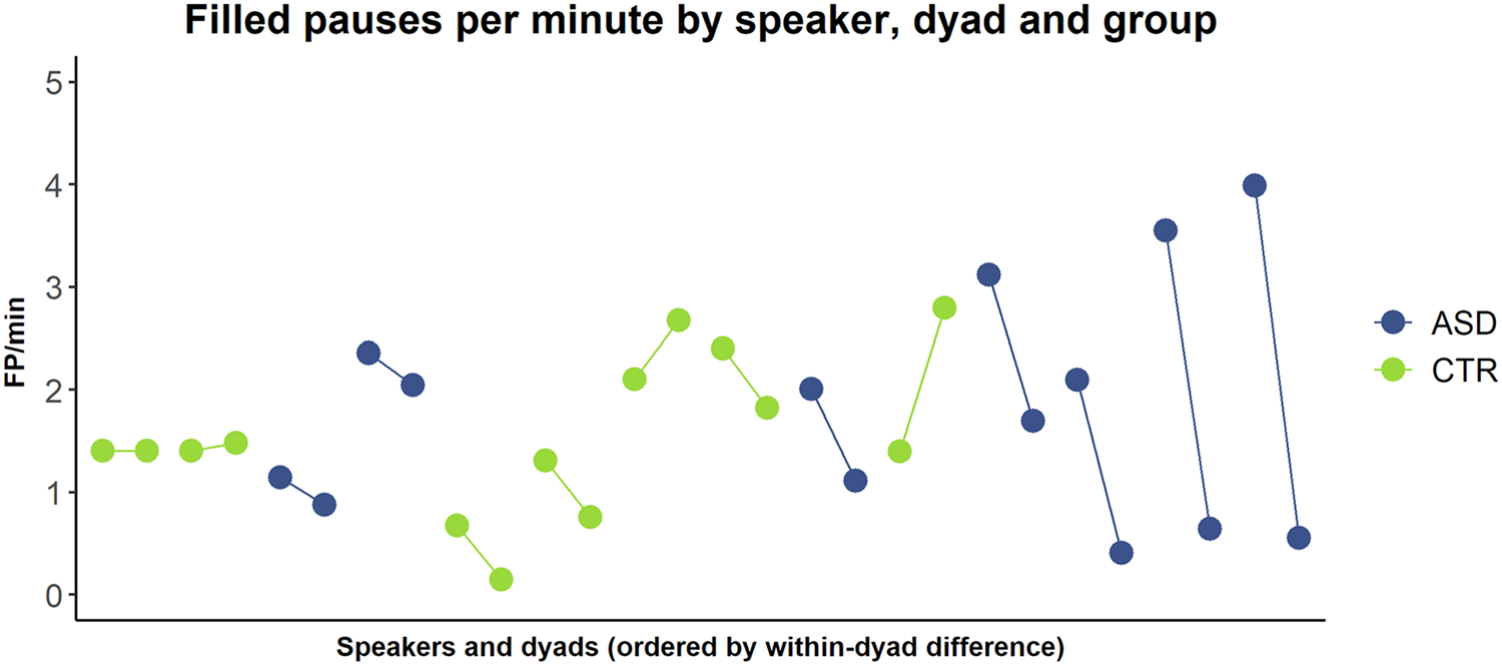
Rate of filled pauses produced per minute of dialogue, by speaker, dyad and group. Speakers within a dyad are connected by lines representing within-dyad differences (by which dyads are ordered on the x-axis). ASD group in blue, CTR group in green

The CTR and the ASD group produced an identical rate of filled pauses per minute (3.63). Underlying this was a very high degree of dyad-specific variability, in both groups, with filled pause rates ranging from 0.82 to 4.82. Furthermore, we found that interlocutors in the ASD group seemed to adapt less to each other within dyads compared to dyads in the CTR group. Specifically, the difference between by-speaker filled pause rates within dyads tended to be much lower in the CTR group (mean = 0.53; SD = 0.44) than in the ASD group (mean = 1.56; SD = 1.18), and ASD dyads also accounted for the 4 greatest within-dyad differences; see Fig. 1.

We used Bayesian negative binomial regression modelling of filled pause rate by dyad to verify that there was no group-level difference. The model output unambiguously confirms this to be the case, with mean δ = − 0.4, 95% CI [− 1.63, 0.78] and *P* (δ > 0) = 0.72.

To test the influence of speaker roles (instruction giver vs. instruction follower) on filled pause production, we calculated proportions, dividing the summed duration of all filled pauses by the summed duration of all speech for givers and followers separately. We calculated proportions instead of rates because speaking times differed considerably between speaker roles.

There was a tendency across groups for instruction givers to produce a higher average proportion of filled pauses (3.71% overall) than instruction followers (2.41% overall). Bayesian modelling taking into account dyad as a random factor suggests that this difference between roles was reliable across groups (for details, see the accompanying repository). Within groups, however, the difference between roles was shown to be reliable only for the ASD group and not for the CTR group. This discrepancy seems to stem from a higher degree of variability in the CTR group. Overall, the behaviour of 9 out of 14 dyads clearly reflected the group level pattern of more and/or longer filled pauses produced by instruction givers.

### Lexical Choice: *uh* Versus *uhm*

**Fig. 2:**
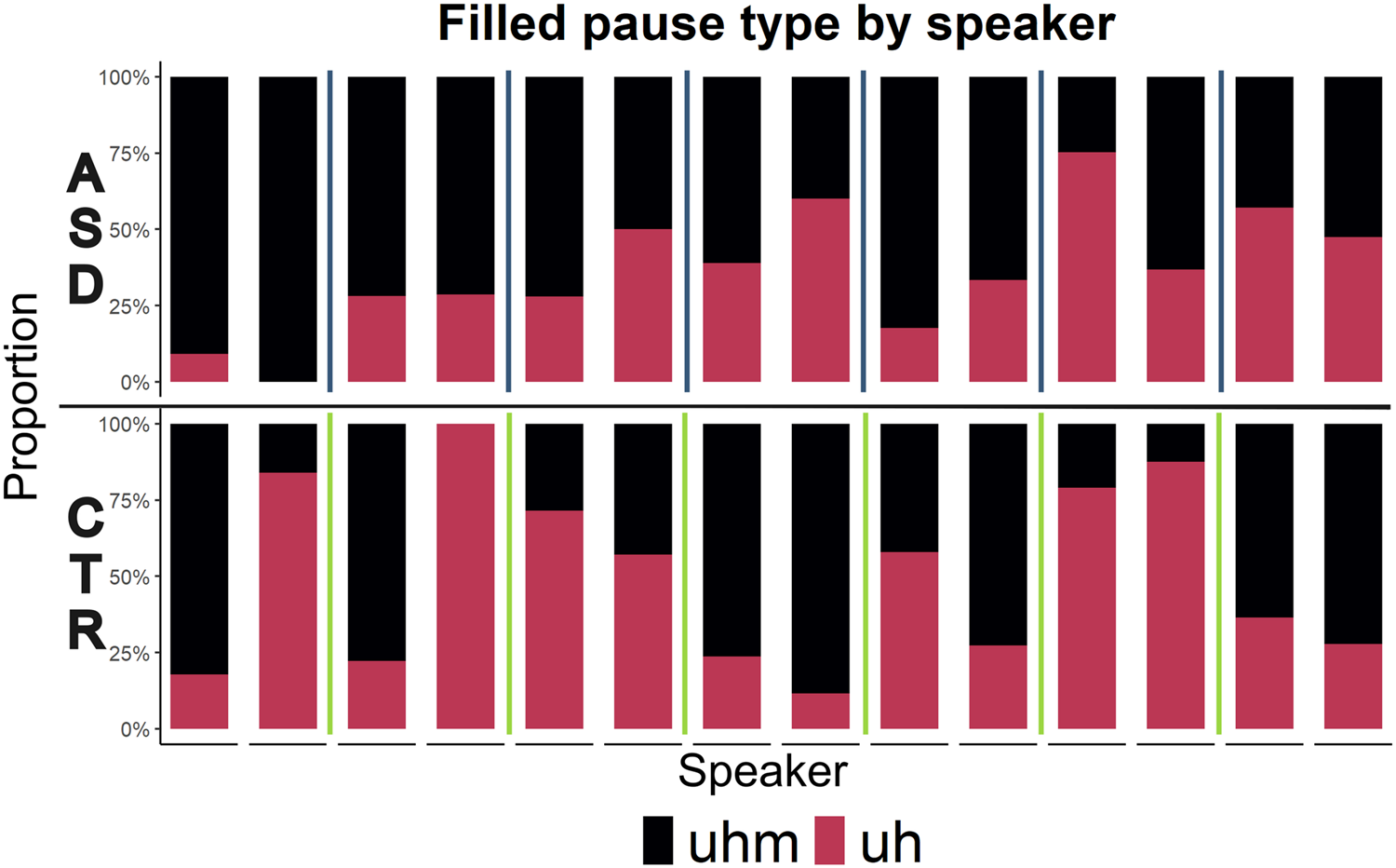
Proportion of filled pause type by group and speaker (as a percentage of their total filled pause productions). *Uhm* (nasal) in black, *uh* (non-nasal) in pink. ASD group in the top row, CTR group in the bottom row. Speakers from the same dyad are plotted next to each other; dyads are separated by vertical lines

Choice of filled pause type was very similar at the group level. Both groups used more *uhm* than *uh* overall, although this preference was slightly stronger for the CTR group (60% *uhm*) than for the ASD group (55.3% *uhm*). This group pattern obscures a very high degree of individual variability, however, with *uhm* proportions ranging from 0 to 100% for different speakers; see Fig. 2. Although fewer CTR speakers showed a preference for *uhm* (7 out of 14) than ASD speakers did (11 out of 14), the preference for one filled pause type over another was not systematic at the group level and instead seems to be a correlate of individual variability.

This high degree of individual specificity combined with the very small initial difference of group averages hence makes it unsurprising that Bayesian modelling of *uhm* proportions by speaker confirms that there was no robust group difference in choice of filled pause type (mean δ = − 4.9; 95% CI [− 15.78, 6.08]; *P* (δ > 0) = 0.77).

### Intonational Realisation

For the prosodic analysis, 176 tokens were discarded because pitch information was not available or was found to be unreliable upon manual inspection (e.g. because tokens were extremely short and/or produced with non-modal voice quality). This left 851 of the original 1027 tokens (82.9%). Note that one speaker (M14, from the CTR group) did not produce any filled pause tokens suitable for prosodic analysis (having produced only 2 filled pauses in total; the average number of filled pauses produced per speaker is 37). Therefore, the following analyses will be limited to the remaining 27 speakers (14 ASD; 13 CTR).

#### Continuous Analysis

A continuous analysis of intonation contours on filled pauses revealed little difference between groups. Both groups produced average values very close to 0 ST, representing little to no pitch movement, i.e. level intonation contours. This is expected according to previous results on the intonational realisation of filled pauses. Mean values were slightly closer to 0 for the CTR group (mean: − 0.29; SD: 1.26) compared to the ASD group (mean: − 0.44; SD: 1.51). Bayesian modelling broadly confirms this trend, but also suggests that it is unlikely to be a robust difference between groups (mean δ = 0.25; 95% CI [− 0.16, 0.67]; *P* (δ > 0) = 0.84).

#### Categorical Analysis

To better account for the special status of level contours (the typical realisation) in the intonation of filled pauses, we further performed a categorical analysis, in which all filled pauses with pitch movement within the range ± 1 ST were categorised as “level”. The tokens exceeding these values were categorised as rises (positive values) and falls (negative values), respectively (cf. Wehrle, 2021; Sbranna et al., 2022).

**Fig. 3:**
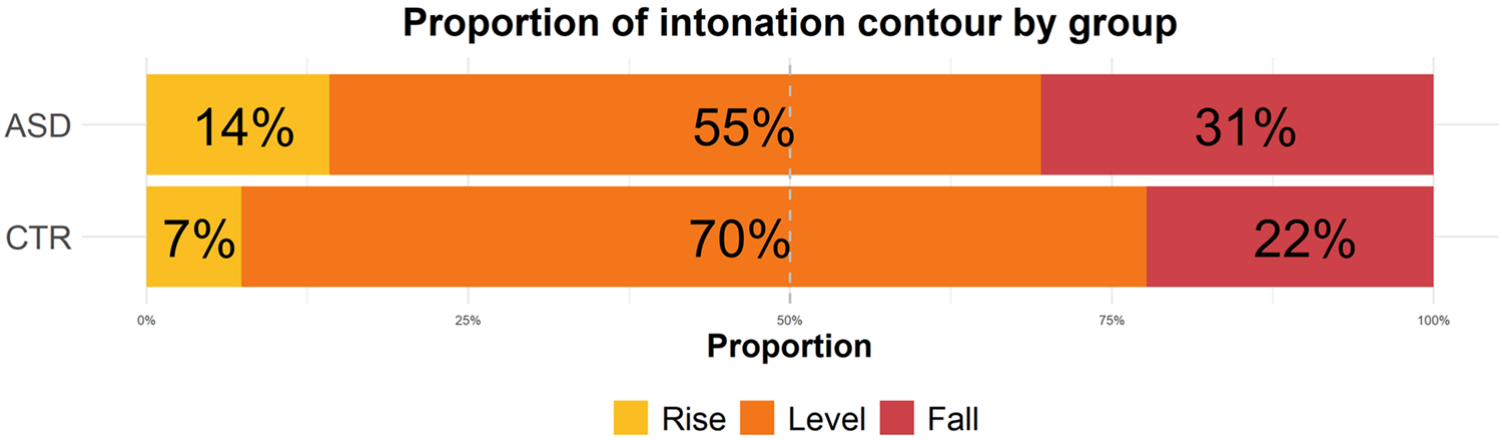
Intonational realisation of filled pauses by group (across type). Rising contours in yellow, level contours in orange and falling contours in red. Level contours were defined as all tokens with a pitch difference in the range of ± 1 semitone

Across filled pause types, the CTR group produced a considerably higher proportion of level contours in the realisation of filled pauses (70.3%) than the ASD group (55.3%), who produced comparatively higher proportions of both rises and falls instead; see Fig. 3. Falling intonation was the second most common realisation in both groups, with rising intonation the least frequent. Bayesian modelling confirms that the group difference in prosodic realisation is robust (mean δ = 12.99; 95% CI [4.28, 21.87]; *P* (δ > 0) = 0.99).

Speaker-specific analysis confirms this pattern in showing, for instance, that 9 out of the 10 lowest proportions of level contours were produced by autistic speakers, whereas the 5 highest proportions of level contours were produced by non-autistic speakers (overall range 23.1–90%).

Comparing the two filled pause types *uh* and *uhm* across groups, we found that *uh* was more often produced with the canonical level contour (70%) than *uhm* (62.1%). Bayesian modelling of the proportions of level contours by filled pause type (*uhm* was the reference level) and speaker (which was treated as a random factor) confirms this as a robust difference (mean δ = 17.05; 95% CI [9.23, 24.82]; *P* (δ > 0) = 1).

Table 2 shows the proportions of level contours used by group and filled pause type. It is clear that level contours constituted the preferred intonational realisation of filled pauses across groups and types (followed by falls, and then rises, which were only rarely used). The pattern is comparatively less obvious for productions by autistic speakers, however. The ASD group produced fewer level contours than the CTR group for both *uhm* and *uh*, but the difference between groups is clearer for *uhm*, as only 49.5% of tokens in the ASD group were produced with a level contour, compared to 68.9% in the CTR group. However, a high degree of by-speaker variability underlies these group-level results and hence, there is no clear effect of the interaction of lexical type and intonation contour in a group-level comparison.

**Table 2:** Proportion of intonation contour by group and filled pause type (level proportions in bold)

Group	Type	Contour	Proportion
ASD	uhm	Fall	32.61%
**ASD**	**uhm**	**Level**	**49.46%**
ASD	uhm	Rise	17.93%
ASD	uh	Fall	27.03%
**ASD**	**uh**	**Level**	**64.86%**
ASD	uh	Rise	8.11%
CTR	uhm	Fall	22.09%
**CTR**	**uhm**	**Level**	**68.90%**
CTR	uhm	Rise	9.01%
CTR	uh	Fall	22.64%
**CTR**	**uh**	**Level**	**72.64%**
CTR	uh	Rise	4.72%

A Bayesian model of proportion of intonation contour by speaker, including the interaction between group and filled pause type and with speaker as a random effect, provides conclusive evidence that (1) fewer level contours were produced by autistic speakers than controls for both *uh* (mean δ = − 14.28; 95% CI [− 25.91, − 1.51]; *P* (δ > 0) = 0.96) and *uhm* (mean δ = − 10; 95% CI [− 19.75, 0.32]; *P* (δ > 0) = 0.95) and (2) that *uh* was produced with a higher proportion of level contours than *uhm* in both the ASD group (mean δ = 16.52; 95% CI [6.47, 26.12]; *P* (δ > 0) = 1) and the CTR group (mean δ = 20.8; 95% CI [9.8, 31.45]; *P* (δ > 0) = 1). Although the difference between groups for intonational realisation was slightly greater for *uhm* compared to *uh*, there is no robust effect for the interaction between group and filled pause type (mean δ = 4.29; 95% CI [-9.26, 17.84]; *P* (δ > 0) = 0.71).

#### Diversity (Entropy)

To quantify how diverse the prosodic realisation of filled pauses was, we used the measure of Shannon entropy as an index of diversity (Shannon, 1948; Wehrle, 2021). The higher the value of entropy (*H*), the more diverse the signal. For instance, in this specific application, the highest possible entropy value is 1.58; this would signify equal proportions for all three types of contours (rising, level and falling). An entropy value of 0, on the other hand, would signify that all filled pauses were produced with the same contour (e.g. level).

In the case of the prosodic realisation of filled pauses, higher entropy values are indicative of more unusual behaviour, as speakers were expected to produce a (very) large proportion of filled pauses with a single intonation contour (level). Based on the results described above, we expected to find higher entropy values for autistic speakers (as they produced fewer level contours).

**Fig. 4:**
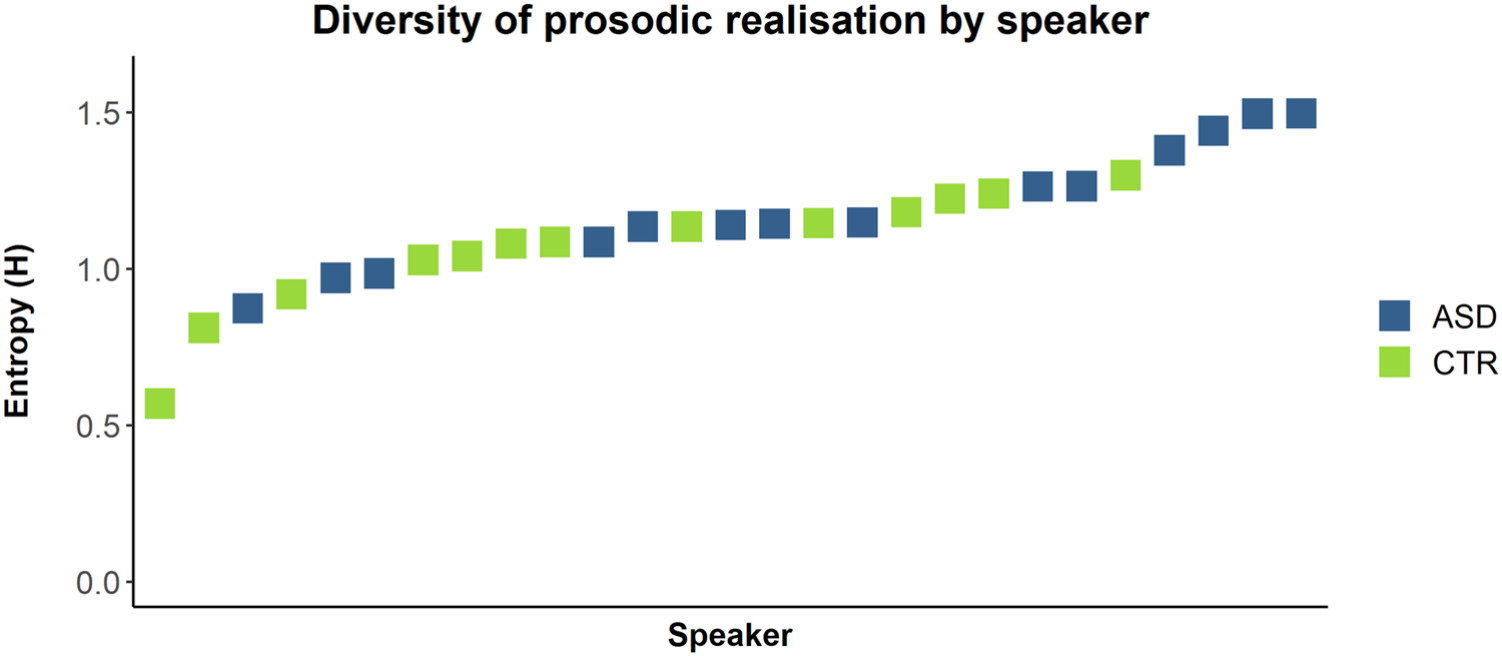
Entropy as a diversity measure of the prosodic realisation (rising, level or falling) of filled pauses, by speaker. Higher entropy values (*H*; on the y-axis) represent a more diverse realisation. ASD group in blue, CTR group in green

Results at the group level indeed reveal a higher entropy value for the ASD group (1.4) compared to the CTR group (1.12). Speaker-specific analysis confirms this pattern as, e.g., 6 out of the 7 highest entropy values were recorded for autistic speakers; see Fig. 4.

Bayesian modelling of entropy values by speaker confirms the group-level difference in the intonational realisation of filled pauses as a robust effect (mean δ = − 0.14; 95% CI [− 0.28, 0]; *P* (δ > 0) = 0.96).

We have to note that entropy operationalised this way does not specifically measure proportions of level contours (as in the preceding section), but rather the diversity of intonation contours used. This means that if a speaker (unusually and unexpectedly) showed a clear preference for a non-level intonation contour (rise or fall), this behaviour would still be represented by a low entropy value. Indeed, 5 out of the 28 speakers in our data set did show a preference for falling instead of level contours in the realisation of filled pauses. However, especially as 4 out of those 5 speakers were part of the ASD group, this does not mitigate the fact that we have shown separate but related evidence for the observations (1) that autistic speakers produced fewer filled pauses with the canonical level contour and (2) that autistic speakers were more diverse in the intonational realisation of filled pauses.

## Discussion

Our results show that autistic and non-autistic speakers did not differ (at all) in the rate of filled pauses produced, nor in their preference of filled pause type (both preferring *uhm* over *uh*). The only group-level difference we found was in prosodic realisation, with ASD speakers producing fewer filled pauses realised with the typical level intonation contour than CTR speakers (although both groups did show a preference for level contours overall). Additionally, interlocutors in the CTR group seemed to adapt more to each other in terms of the rate of filled pauses produced compared to the ASD group. It is also interesting to note that the more frequent lexical type *uhm* was less consistently produced with a level contour, across groups, although this could simply be related to the fact that *uhm* was, on average, almost twice as long as *uh.* This increase in duration might in itself have led to the production of more falling contours (Fuchs et al., 2015; Gussenhoven & Rietveld, 1988; Wehrle et al., 2023a).

While ours is the first study to analyse prosodic aspects of filled pause production in ASD, we can compare our results on rate and lexical choice with previous studies on these aspects. Superficially, the fact that we did not find any differences in filled pause rate or preference of type (*uhm* over *uh*) supports, perhaps surprisingly, the findings from only one study (Suh et al., 2014) and stands in contrast to the other relevant findings (Gorman et al., 2016; Irvine et al., 2016; Jones et al., 2022; Lake et al., 2011; Lunsford et al., 2010; McGregor & Hadden, 2020; Parish-Morris et al., 2017).

A direct comparison with our results, however, is not possible as none of the previous studies investigated semi-structured conversations between autistic adults, instead tending to focus on speech elicited in more highly structured, formal contexts and produced by children (usually interacting with non-autistic adults). A related issue is the inclusion of (autistic and non-autistic) speaker groups with a very wide age range in previous work, leading to one such sample being described as “children from 8 to 21 years old” (Suh et al., 2014, p. 1684).

Findings from the only other study investigating filled pause productions by autistic adults (Lake et al., 2011) crucially differ from our own. We did not find any difference in filled pause rate, whereas this earlier study found a lower rate for both *uh* and *uhm* in their ASD group. At the same time, there is an important similarity between this previous study and our own, as in both cases there is no evidence for a special role of *uhm*, in particular, for distinguishing the behaviour of autistic and control subjects (in contrast to all the studies on autistic children mentioned above). While we do not wish to speculate widely about causes and implications on the basis of two studies, it does seem plausible (1) that the role of *uhm* being more listener-oriented compared to *uh* may have been exaggerated in some previous research, at least where such conclusions were drawn on the basis of the fact that some autistic speakers seemed to produce *uhm* less often than control speakers, and (2) that continuous development and successful social camouflaging might play important roles in autistic adults behaving more similarly to their non-autistic peers than is the case for children.

More generally, as filled pauses are most prevalent and functionally important in conversational interaction (Corley & Hartsuiker, 2003; Fox Tree, 2001), the external validity of results based on speech elicited through, e.g., highly structured interviews with children (Gorman et al., 2016), picture story narrations (Suh et al., 2014) or descriptions of a series of paintings with the added task of simultaneously tapping an index finger as fast as possible (Irvine et al., 2016) has to be questioned. Speculations as to the pro-social nature of filled pauses are similarly problematic when they are founded on this kind of speech data. Engelhardt et al. (2017) rightly point out some important issues in the interpretation of conversational behaviours as being either speaker- or listener-oriented in such contexts (and also criticise the fact that previous research did not appropriately account for individual differences). Somewhat puzzlingly, Engelhardt et al. then proceed to describe production data from a sentence-repetition task, which did not yield a single filled pause token (as might be expected, partly because there is no need in this context to use filled pauses to facilitate the planning of an utterance).

Of course, our own work also has a number of important limitations. We investigated task-based, rather than fully free conversations. Our experimental setup also limited interlocutors to the spoken modality, thus excluding gesture and eye gaze behaviours that are functionally equivalent to spoken filled pauses (Beattie, 1979; Brône et al., 2017; Kosmala & Morgenstern, 2017). Despite these constraints, we are confident that the elicitation method used here constitutes an improvement over those used in related studies and described above, foremost because it enables us to analyse social *interactions* between *disposition-matched* interlocutors (cf. Dingemanse et al., 2023). From a methodological standpoint, we acknowledge that we are not able to provide a qualitative analysis taking into account e.g. conversational context and functions of (different kinds of) filled pauses in this work, but we are planning to address these important issues in follow-up studies. A more specific methodological limitation concerns the prosodic analysis. Here, we calculated the difference in pitch between two fixed time points, near the beginning and the end of each token. Because filled pauses are very short (< 500 ms in almost all cases), this somewhat simplified view does capture the essential qualities of intonation contours and is perceptually valid. Nevertheless, and although our data set does not contain any tokens with a clear inflection point in the middle of the pitch contour (i.e. complex contours such as rise-falls), future investigations should explore the use of more fine-grained techniques such as polynomial modelling (Belz & Reichel, 2015), generalised additive mixed modelling (Sóskuthy, 2021) or analyses in the ProPer framework (Albert et al., 2018, 2020; Cangemi et al., 2019). Finally, we investigated a limited sample of subjects from one extreme end of the autism spectrum (verbal, socially relatively skilled and motivated individuals with average or above-average IQ). Our data do not allow us to generalise the present findings to interactions between disposition-mixed dyads (ASD–CTR) or to fully spontaneous, multi-modal interaction. On the other hand, this specific limitation could also be argued to add to the specificity and interpretability of the results presented here.

Moving beyond issues of comparability and methodology, the fact that we did not replicate the previous finding that filled pauses are produced at a lower rate in ASD, or that nasal filled pauses (*uhm*) are dispreferred, seems to us reason enough to call into question (1) the causal interpretation of filled pauses as specifically and exclusively “other-directed” signals (e.g. Lake et al., 2011) and (2) the appropriateness of using characteristics of filled pauses, specifically the production of *uhm*, as a pragmatic or clinical marker for ASD, as has been suggested in previous work (Irvine et al., 2016; McGregor & Hadden, 2020). In general, the use of *uhm* might well differ from that of *uh* in important and general ways. For instance, it has been shown that silences following *uhm* are longer than silences following *uh* (Clark & Fox Tree, 2002), and we recently replicated and extended this result for both autistic and non-autistic speakers (Wehrle et al., 2023a). However, just as we did not find differences between the ASD and the CTR group in that study, both the results presented here and in the previous study by Lake et al. (2011) suggest that, while the use of *uhm* may differ between autistic and non-autistic children, this is not necessarily the case for adult speakers.

## Conclusion

The results presented in this work deepen our understanding of filled pause use by speakers on the autism spectrum specifically, but also have broader implications for the study of conversation in ASD. The fact that our study is not directly comparable with any previous work highlights the importance of four aspects (and their interactions) that have often been neglected to date: (1) studying disposition-matched (ASD–ASD) in addition to mixed dyads, (2) studying the behaviour of autistic adults and understanding it as potentially distinct from that of autistic children, (3) studying prosodic aspects of conversational behaviour in ASD and (4) moving to more ecologically valid settings in the elicitation of speech data. Regarding the experimental findings presented here, a particularly valuable (and novel) observation is that autistic speakers showed differences in the prosodic realisation of filled pauses. This intriguing result is likely to be salient and impactful in spoken interaction, as previous work on closely related discourse markers (backchannels) has shown that their precise prosodic realisation can have an impact on listener judgements and mutual understanding. Testing such assertions empirically in the context of filled pauses opens up a highly promising avenue for future investigations, which should also aim to include perception experiments, multi-modal analyses, comparisons between male and female speakers, and qualitative analyses of functional aspects of filled pause use in different conversational contexts. Additionally, our result on reduced within-dyad adjustment in terms of filled pause rate in the ASD group points to the importance of an in-depth investigation and quantification of conversational alignment in future work.

### Supplementary Information

Below is the link to the electronic supplementary material.

Supplementary file1 (DOCX 13 kb)

## Data Availability

All data and scripts are available in the OSF repository at https://osf.io/6zu4g/. The data folder contains csv files with the experimental data and subject data. The main folder contains an RMarkdown file (in .rmd and .html formats) in which the entire manuscript is reproduced with code chunks that were used to produce all plots and perform all modelling, placed directly adjacent to the relevant portions of the manuscript.
