# Supplementary material for: Filled Pauses Produced by Autistic Adults Differ in Prosodic Realisation, but not Rate or Lexical Type
Source: J Autism Dev Disord. 2023 May 3;54(7):2513–25. doi: 10.1007/s10803-023-06000-y (PMC11286642; doi:10.1007/s10803-023-06000-y)
Supplement: Supplementary file 1 — Supplementary file1 (DOCX 13 kb) [file 10803_2023_6000_MOESM1_ESM.docx]

**S1 Table. Dialogue duration, number of filled pauses (FP), FP type and FP duration, by group.**

| Group | Mean dialogue duration (SD) | Total FPs | Total *uhm* (proportion) | Total *uh* (proportion) | Mean FP duration (SD) |
| --- | --- | --- | --- | --- | --- |
| ASD | 14’ 37’’  (7’ 12’’) | 365 | 202 (55.3 %) | 163 (44.7 %) | 423 ms (242) |
| CTR | 26’ 01’’  (14’ 35’’) | 662 | 397 (60 %) | 265 (40 %) | 456 ms (249) |
| Total | 20’ 19’’ (12’ 32’’) | 1027 | 599 (58.3 %) | 428 (41.7 %) | 444 ms (247) |
